# GWO-Based Joint Optimization of Millimeter-Wave System and Multilayer Perceptron for Archaeological Application

**DOI:** 10.3390/s24092749

**Published:** 2024-04-25

**Authors:** Julien Marot, Flora Zidane, Maha El-Abed, Jerome Lanteri, Jean-Yves Dauvignac, Claire Migliaccio

**Affiliations:** 1 Centrale Mediterrannée, CNRS, Aix Marseille Université, Institut Fresnel, 13397 Marseille, France; julien.marot@fresnel.fr; 2 Universite Cote d’Azur, Laboratoire d’Electronique, Antennes et Telecommunications (LEAT), Campus SophiaTech, Bât. Forum, 930 Route des Colles— BP 145, 06903 Sophia Antipolis, France; flora.zidane@univ-cotedazur.fr (F.Z.); maha.el-abed@univ-cotedazur.fr (M.E.-A.); jerome.lanteri@univ-cotedazur.fr (J.L.); jean-yves.dauvignac@univ-cotedazur.fr (J.-Y.D.)

**Keywords:** radar system, GWO, co-design, classification

## Abstract

Recently, low THz radar-based measurement and classification for archaeology emerged as a new imaging modality. In this paper, we investigate the classification of pottery shards, a key enabler to understand how the agriculture was introduced from the Fertile Crescent to Europe. Our purpose is to jointly design the measuring radar system and the classification neural network, seeking the maximal compactness and the minimal cost, both directly related to the number of sensors. We aim to select the least possible number of sensors and place them adequately, while minimizing the false recognition rate. For this, we propose a novel version of the Binary Grey Wolf Optimizer, designed to reduce the number of sensors, and a Ternary Grey Wolf Optimizer. Together with the Continuous Grey Wolf Optimizer, they yield the CBTGWO (Continuous Binary Ternary Grey Wolf Optimizer). Working with 7 frequencies and starting with 37 sensors, the CBTGWO selects a single sensor and yields a 0-valued false recognition rate. In a single-frequency scenario, starting with 217 sensors, the CBTGWO selects 2 sensors. The false recognition rate is 2%. The acquisition time is 3.2 s, outperforming the GWO and adaptive mixed GWO, which yield 86.4 and 396.6 s.

## 1. Introduction

About 13,000 years ago, humanity moved from nomad to sedentary life styles in the fertile crescent. In the 9000 years Before Common Era (BCE), men started to raise animals, to cultivate, and to make pottery for food storage [1]. How agriculture spread from Mesopotamia to the rest of the world is still a subject of study for Historians, made all the more challenging by the fact that writing emerged two thousand years later, about 3400 BCE. Looking for pottery techniques is one of the key enablers for solving this issue. Indeed, historians found that the coiling technique has spread through Central and East Europe, whereas the spiral one follows the Western and Mediterranean routes. Of course, the remains that can be found by archaeologists are not entire potteries but shards, which poses a new issue: how to recognize the pottery technique based on small pieces of potteries? We have recently proposed a new imaging modality to solve this issue [2]. It is based on low-THz radar-based Non-Destructive Evaluation (NDE) and classification with a neural network. We emphasize, in [2], the interest of millimeter waves for this research. Low-THz waves adequately capture pieces of information about the content of an archeological shard, in particular the shape of the pores inside the shards, thanks to the high resolution due to the short wavelength. The classification accuracy is close to 100%, which is very promising for quite a number of applications. However, in its present form, the measurement time (about 2 h per shard) hinders its extension to an application that requires real-time operation. This is a well-known problem in many radar-based applications. On one hand, the more spatial diversity we have, the better the image. At the same time, the measurement sampling has to be dense in order to avoid spurious responses. This leads to a significant number of measurements points (number of sensors), which in turn slows down the measurement. These constraints are essentially based on the need to recognize the object for imaging-based identification. In this paper, we propose to overcome this issue with a co-design scheme. To this end, we are implementing a joint optimization of the measurement system and the parameters of the Multi-Layer Perceptron (MLP) used for the classification. Our main objective is to drastically reduce the number of antennas (measurements points) for reaching real-time operation. In [3], sensor placement is optimized in a wireless sensor network with a step-wise optimization approach; but optimizing the placement of the radar sensors independently of each other is a brand new outcome.

The interest of neural networks has been demonstrated to process multivariable sensor data [4], or radar data with the aim of human activity classification (walking, running, etc.) [5]. They also have been adapted to the design of radiofrequency and microwave antennas [6,7,8], noise modeling in transistors [9], optimization of circuits [10], and to locate fault elements in arrays of sensors [11].

The main contributions of this paper are as follows. A careful look at the parameters involved in the considered radar issue permits to distinguish three types of ’search spaces’, each containing all the possible values for a given parameter. For instance, the state of a given sensor corresponds to a binary search space: either 0, or 1, standing for OFF or ON. The other parameters that are investigated correspond to a continuous search space and a ternary search space. To tackle this issue, we propose a novel optimization algorithm, inspired by the GWO (Grey Wolf Optimizer), which we call the CBTGWO (Continuous Binary Ternary Grey Wolf Optimizer), to minimize a criterion which depends on a false recognition rate and on the number of sensors. Firstly, we derive a variant of the Binary GWO which favors the reduction in the number of sensors which are switched on, and a Ternary GWO. Secondly, we embed chaotic sequences in the update rules to enhance the exploration and exploitation abilities of our algorithm. We propose an original way to profit by the diversity of chaotic sequence, which exhibits the great advantage of authorizing a perfect control of the behavior of the algorithm in the last iterations of the process. The results obtained on either a single frequency context or on a wide-band context show the ability of the proposed method to drastically reduce the number of sensors while offering small false recognition rate values.

In Section 2, we provide the mathematical background and state-of-the-art about the fundamentals of the GWO, and its binary version in particular. We explain what the materials used in this paper are, such as the radar data used for shard classification. We present the millimeter-wave system for shard analysis, and the neural network algorithm, which is meant for data classification. We set the problem we wish to solve: the common minimization of a false recognition rate and a number of sensors which are switched on. We derive a single criterion that combines these two criteria, and we show that the parameters which have an influence on this criterion belong either to discrete, ternary, or binary search spaces. In particular, each sensor state corresponds to a binary search space: either ON, or OFF.

In Section 3, we detail the novel method proposed in this paper. Firstly, a novel binary version of the GWO, which aims at estimating the optimal state of the sensors while favoring 0 values, and enhances exploration thanks to an evolutive update rule. Secondly, the combination of the continuous, binary, and ternary versions of the GWO yields the CBTGWO.

In Section 4, we evaluate the performances of the CBTGWO, compared to the vanilla GWO [12] and the adaptive mixed GWO [13]: firstly, on a synthetic ’surrogate’ function, which models the practical radar issue under study, and, secondly, on the considered issue and real-world radar experimental data. A discussion about these results is provided in Section 5, and conclusions are drawn in Section 6.

We use these notations in the paper:


Manifoldblackboard bold

A

Matricesboldface upper-case roman
**A**
Vectorsboldface lower-case roman
**a**
Scalarslower-case or upper-case romana, *a* or *A*


A vector **a** with *P* scalar components can be expressed as $a={\left[a^{1},a^{2},\ldots ,a^{P}\right]}^{T}$.

We use these definitions in the paper:


Vector with *P* components


$a={\left[a^{1},a^{2},\ldots ,a^{P}\right]}^{T}$

Hadamard product∘$a\circ b={\left[a^{1}b^{1},a^{2}b^{2},\ldots ,a^{P}b^{P}\right]}^{T}$.Absolute value


|·|

Interval

[a:b]

*a* and *b* are real scalar valuesSet of discrete values

$\left\{a,\ldots ,b\right\}$

*a* and *b* are real scalar values


## 2. Theoretical Background and Materials

In this section, we present the theoretical background, which is necessary to understand the novel algorithm that we have developed to solve the considered issue, and the materials used in this paper.

In Section 2.1, we remind the background about various metaheuristics and the vanilla GWO algorithm, and we focus on discrete and binary versions of the GWO. Section 2.2 details the acquisition of radar data and the classification method. This subsection includes a general overview of metaheuristic algorithms, and focuses on different versions of the GWO.

### 2.1. Theoretical Background

#### 2.1.1. Background on Metaheuristic Algorithms

Metaheuristic algorithms have demonstrated their capacity in finding optimal solutions in the frame of various issues in soft computing. One of these issues, which attracts much attention since the increasing interest for neural networks, is the parameter tuning of these networks. Referring to [14], the number of journal papers dealing with neural networks is up to 12,000 in 2018 (4000 in 2015); and the number of papers dealing with neural networks which are optimized with metaheuristics increases from 400 to 600 in the same period. Therefore, it seems that parameter tuning of neural networks starts attracting attention but still concerns a limited amount of studies. Among others, in [15], a compressed version of the VGG-16 (Visual Geometry Group-16) convolutional neural network is obtained through an optimization of its structure with a modified version of the coral reef optimization algorithm. In [16], an optimal network for face recognition is designed with a rider optimization algorithm, which tackles high noise and occlusion. This algorithm optimizes the number of convolutional layers, pooling layers, fully connected layers, hidden layers, and types of activation function and pooling. It is worth noticing that there are five possible values for the activation function and three for the pooling.

In [17], a GWO algorithm is applied as a global search method to determine the weights of a MultiLayer Perceptron (MLP). In [18], a new hybrid wind speed forecasting model is developed based on Long Short-Term Memory (LSTM) networks. The GWO is adopted to determine eleven LSTM model weights in a continuous search space. In the field of image processing, autoencoders have attracted attention, as they preserve the dimensionality of the data: when the input is an image, the output is also an image. Optimized autoencoders, for instance, have already been applied to synthetic aperture radar image processing [19,20,21], and to hyperspectral images [22]. Some improved versions of the GWO have been applied to path planning [23,24].

Parameter tuning is carried out with a grid search, except in [21] where a multiobjective metaheuristics is used for this purpose. In [25], a stacked and sparse denoising autoencoder is proposed to reduce the wall clutter in indoor radar images. The results are convincing but the parameter tuning issue is not considered at all.

In [26], the automatic tuning of hyperparameters of an MLP is performed with the GWO to identify COVID-19-affected chest X-ray scans. Still for a medical application, in [27], a cellular genetic algorithm is designed with a special crossover operator to optimize weights and biases of the MLP to classify medical data.

A first remark is that the metaheuristics cited above estimate optimal hyperparameters of neural networks in continuous search spaces. This might not be the most appropriate way to choose between a reduced number of possibilities, such as three types of solvers. A second remark is that, to the best of our knowledge, few papers investigate any application where a joint estimation is performed for the parameters related to the acquisition and to the processing with neural networks.

#### 2.1.2. Background on the Grey Wolf Optimizer

The seminal work of Mirjalili [12] is based on the observation of wolves. Equations have been derived which mimic the behavior of wolf herds, and model their displacements, depending on their role in the hierarchy of the herd. The Grey Wolf Algorithm (GWA) aims at modeling the displacement of all members of the herd towards a ‘prey’ which represents the global minimum of the considered objective function, in an iterative agent-based algorithm. The algorithm assumes that *P* parameters are expected: $K^{1},K^{2},\ldots ,K^{i},\ldots ,K^{P}$, where P≥1. The following notations hold: *P* is the number of expected parameters, which are indexed with *i*; iter denotes one iteration and $T_{max}$ the total number of iterations; and C(·) is the objective function, also called criterion, which depends on the *P* parameters. In this paper, unless specified, minimization problems are considered.

Wolves are represented through vectors: $x_{q}(iter)$ models a wolf indexed by q=1,…,Q at iteration iter. It contains the following values: $x_{q}(iter)={\left[K^{1},K^{2},\ldots ,K^{P}\right]}^{T}$.

The vanilla version of the GWO searches a continuous space [12]. The hierarchy of the herd divides it into the three leaders α, β, and δ, and the other agents which are called the ω wolves: $x_{\alpha }\left(iter\right)$, $x_{\beta }\left(iter\right)$, and $x_{\delta }\left(iter\right)$ denote the position of the leaders α, β, and δ, respectively, at iteration iter. The position of any wolf at iteration iter+1 is calculated as 

(1) $$x_{q}(iter+1)=\frac{y_{\alpha }\left(iter\right)+y_{\beta }\left(iter\right)+y_{\delta }\left(iter\right)}{3}$$

 It results from the equal contribution of the α, β, and δ wolves. These contributions are computed at each iteration iter as follows, for any leader *l*, either α, β, or δ:

(2) $$y_{l}(iter)=x_{l}(iter)-\Delta_{x}\left(iter\right)$$

where $\Delta_{x}\left(iter\right)$ is a random additional term which decreases to 0 across iterations. It is defined as follows:

$\Delta_{x}\left(iter\right)=b\circ d_{l}$ where $d_{l}(iter)=|c\circ x_{l}(iter)-x_{q}(iter)|$.

The vectors **b** and **c** are calculated as $b=2a\circ r_{1}-a$ and $c=2r_{2}$. In these expressions, vectors $r_{1}$ and $r_{2}$ have random components between 0 and 1. For the sake of a perfect understanding of the rest of this paper, we detail below the component-wise notations of these update rules for each parameter i=1,…,P:

The component $b^{i}$ of **b** is defined as

(3) $$b^{i}=2ar_{1}-a,$$

The component $d_{l}^{i}(iter)$ of $d_{l}(iter)$ is defined as

(4) $$d_{l}^{i}(iter)=|2r_{2}x_{l}^{i}-x_{q}^{i}\left(iter\right)|$$

where $r_{1}$ and $r_{2}$ are two random values between 0 and 1; $x_{q}^{i}\left(iter\right)$ is the *i*th component of the *q*th agent at iteration iter; and $x_{l}^{i}\left(iter\right)$ is the *i*th component of leader *l*.

The component $y_{l}^{i}\left(iter\right)$ of $y_{l}\left(iter\right)$ is defined as

(5) $$y_{l}^{i}\left(iter\right)=x_{l}^{i}-b^{i}d_{l}^{i}\left(iter\right)$$

With the notations above, Equation (1) can be expressed as

(6) $$x_{q}^{i}\left(iter+1\right)=\frac{y_{\alpha }^{i}\left(iter\right)+y_{\beta }^{i}\left(iter\right)+y_{\delta }^{i}\left(iter\right)}{3}$$

The hunting process is divided into two phases: during the ‘exploration’ the wolves look for the prey, and during the ‘exploitation’ they kill the prey. To distinguish between these phases, the value of *a* in Equation (3) takes the following form:

(7) $$a=2(1-\frac{iter}{T_{max}})$$

Different expressions of *a* have been proposed in various versions of the GWO, either ‘modified’ [28], ‘adaptive’ [13], or including chaotic sequences [29]. In all these versions, when a>1 (exploration phase), wolves may diverge from the leaders; and when a≤1, wolves converge towards the leaders.

#### 2.1.3. Discrete, Binary, and Ternary Grey Wolf Optimizers

The mixed and adaptive mixed GWO proposed in [13] handle jointly continuous and discrete search spaces. They combine a continuous version and a discrete version of the GWO. The discrete version handles all types of discrete search spaces, including binary, but it is not specifically meant for binary spaces. Some other methods are specifically dedicated to binary spaces. In [30], a Binary GWO is proposed where values in a continuous space are turned to binary with a rounding operation. In [31,32], a selection is made which rules the evolution of the wolves. This selection process replaces the wolves’ movements in the seminal version of the GWO [12], and their discrete displacements proposed in [13]. Here are some mathematical details about the process, described in [31], for the selection of the updated value of a wolf in a binary search space.

Firstly, we define

$$\varphi :\;\left[0:1\right]\times R_{+}\times R_{+}\to \left[0:1\right];\;\left(y,c1,c2\right)\mapsto \varphi \left(y,c1,c2\right)$$

(8) $$\varphi (yc1c2)=\frac{1}{\sqrt{1+exp(-c1(y-c2))}}$$

where c1 and c2 are parameters in R.

**Figure 1:**
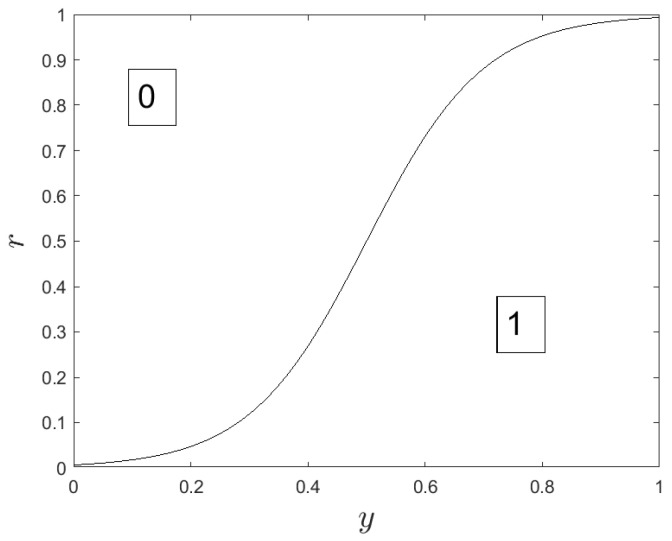
Binary map.

φ is usually called ‘Sigmoid’ and exhibits an inflection point at location c2. In the following, *r* is a real random number between 0 and 1. In dimension *i* (i=1,…,N), wolf *q* is updated from iteration iter to iteration iter+1 as follows:

$x_{q}^{i}\left(iter+1\right)=$

(9) $$\;\left\{\begin{array}{ccc} 1 & if & r\leq \frac{y_{\alpha }^{i}\left(iter\right)+y_{\beta }^{i}\left(iter\right)+y_{\delta }^{i}\left(iter\right)}{3}10,0.5\\ 0 & otherwise \end{array}\right.$$

where $y_{\alpha }^{i}\left(iter\right)$, $y_{\beta }^{i}\left(iter\right)$, and $y_{\delta }^{i}\left(iter\right)$ represent the contribution, in dimension *i*, of leaders α, β, and δ. The computation of these contributions is presented below in Equation (10). In Figure 1, we display the binary map used in Equation (22) to choose either $x_{q}^{i}\left(iter+1\right)=0$ or $x_{q}^{i}\left(iter+1\right)=1$.

In Equation (22), the weighted contribution $\frac{y_{\alpha }^{i}+y_{\beta }^{i}+y_{\delta }^{i}}{3}$ of the leaders is the input of a transform function: the larger this contribution, the more probable the selection of the value 1 as an updated value $x_{q}^{i}\left(iter+1\right)$.

The contribution $y_{l}^{i}\left(iter\right)$ for any leader *l* (either α, β, or δ), in dimension *i*, is computed as follows:

(10) $$y_{l}^{i}\left(iter\right)=\left\{\begin{array}{ccc} 1 & if & (x_{l}^{i}+bstep_{l}^{i})\geq 1\\ 0 & otherwise \end{array}\right.$$

where

(11) $$bstep_{l}^{i}=\left\{\begin{array}{ccc} 1 & if & cstep_{l}^{i}\geq r\\ 0 & otherwise \end{array}\right.$$

where

(12) $$cstep_{l}^{i}=\varphi (b^{i}d_{l}^{i}(iter)10,0.5$$

In Equation (12), $b^{i}$ is the *i*th component of vector **b**; and $d_{l}^{i}(iter)$ the *i*th component of vector $d_{l}(iter)$, *l* denoting the considered leader.

In [29], a ternary update rule has been proposed which selects either 0, 1, or 2, in a similar manner as in Equation (22), but with a ternary map involving two functions $\varphi^{u}$ and $\varphi^{d}$:  

(13) $$x_{q}^{i}\left(iter+1\right)=\left\{\begin{array}{ccc} 0 & if & r\geq \varphi^{u}\left(\frac{y_{\alpha }^{i}(iter)+y_{\beta }^{i}(iter)}{2},a\right)\\ 1 & if & r<\varphi^{u}\left(\frac{y_{\alpha }^{i}(iter)+y_{\beta }^{i}(iter)}{2},a\right)\;and\;r\geq \varphi^{d}\left(\frac{y_{\alpha }^{i}(iter)+y_{\beta }^{i}(iter)}{2},a\right)\\ 2 & if & r<\varphi^{d}\left(\frac{y_{\alpha }^{i}(iter)+y_{\beta }^{i}(iter)}{2},a\right) \end{array}\right.$$

where the scalar *r* is a random value between 0 and 1 and taken from a normal distribution. Function $\varphi^{u}$ separates the uppermost part of the map from the rest of the map; and function $\varphi^{d}$ separates the lowermost part of the map from the rest of the map.

Section 2.2 presents the considered application: the radar sensor system and the neural network dedicated to aracheological shard classification.

### 2.2. Materials: Millimeter-Wave Radar Acquisitions and a Multilayer Perceptron for Shard Classification 

We consider an active radar acquisition system which transmits and receives millimeter waves, using a certain amount of sensors. In a previous work, the millimeter-wave measurements were processed with a two-dimensional fast Fourier transform to generate real-valued data [2]. In this paper, we use the measured complex data directly. In addition, we no longer aim at using a regular sensor sampling: the sensors are no longer supposed to be regularly spaced eventually. They can be switched on or off independently from each other.

#### 2.2.1. Description of the Neural Network

**Figure 2:**
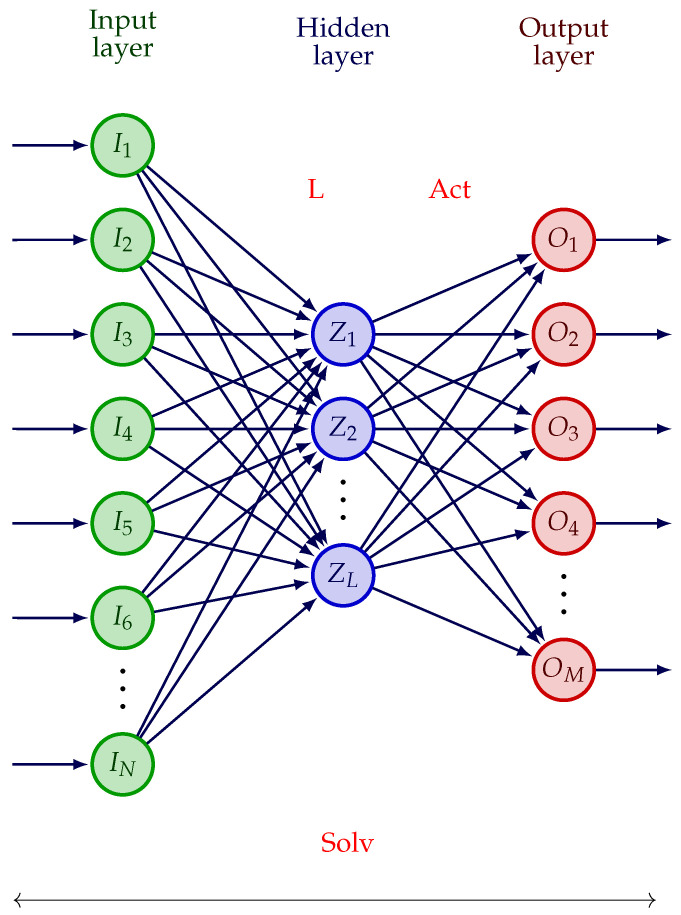
One hidden layer-MLP used for shard classification. ‘Solv’ denotes the solver, ‘L’ denotes the number of neurons in the hidden layer, ‘Act’ denotes the activation function associated with the neurons at the output layer.

We tackle a classification issue: with a neural network called multilayer perceptron [33], we wish to distinguish between two types of archaeological shards. So, our purpose is twofold: we wish to use the least possible number of sensors and, at the same time, to reach the best classification performances. For this, the proposed algorithm should select the least possible number of sensors and place them in the best manner, and it should tune the parameters involved in the multilayer perceptron. This ‘co-design’ consists in tuning the parameters involved in both data acquisition and processing jointly, because all of them influence the false recognition rate, which should be the smallest possible. Our aim is to develop an algorithm which will be applied to the selection of the relevant parameter values in this radar-based non-destructive testing workflow. Figure 2 represents the structure of the MLP that we use in our application. It includes one hidden layer. The number of input neurons *N* is equal to the number of elements in the sample millimeter-wave signals, that is, a number of complex values. The values of any sample signal are denoted by $I_{n}$, n=1,…,N. The number of neurons in the hidden layer is denoted by *L*. Each output neuron is denoted by $O_{m}$, m=1,…,M.

In our classification issue, we aim at classifying the shards into coil or spiral. We therefore deal with a binary classification problem. Two classes are considered, and the number of output neurons *M* is equal to 1: the value of this single output neuron is either 0 or 1. There are three main hyperparameters to tune in this network: the activation function (Act.), the solver (Solv.), and the number of neurons on the hidden layer (*L*). *L* is generally higher than the number of classes *M* and influences the classification performances. In the MLP that we use, the activation function is the same for all neurons. In this work, we will use the GWO-based algorithms to optimize the three hyperparameters mentioned above.

#### 2.2.2. Problem Setting: Description of the Radar System and the Dataset

**Figure 3:**
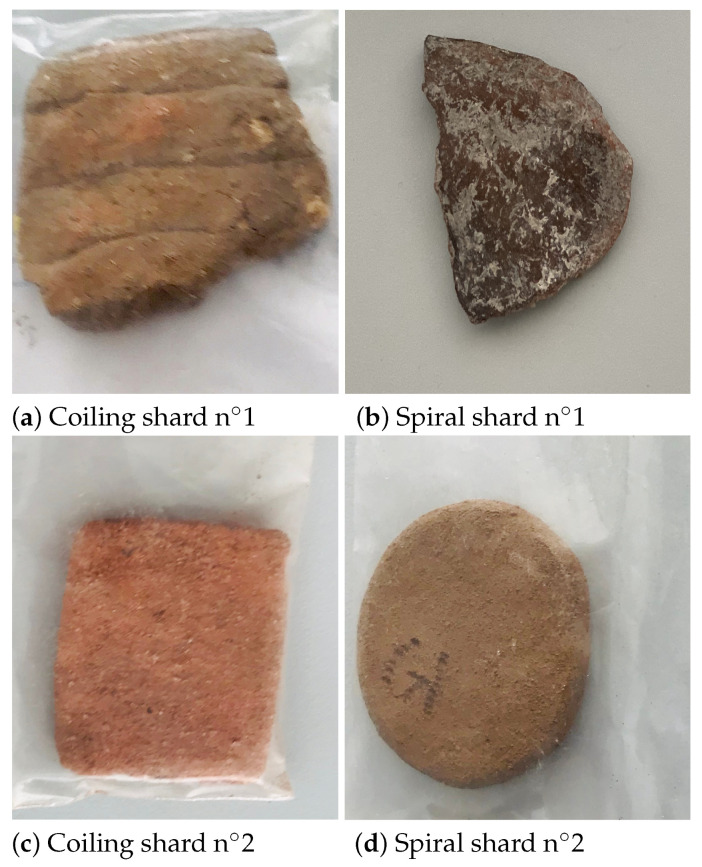
Archaeological samples: coiling and spiral shards.

As mentioned in the introduction, archaeologists have studied the introduction of agriculture in Europe during the 6th and 7th millennia Before Common Era based on the study of pottery techniques. They have shown that the introduction of agriculture followed two routes: the Central European one characterized by the technique of the coil, while the Mediterranean route is associated with the technique of the spiral. The two techniques are distinguished by the air bubbles (pores) formed during the manufacturing process [34]. In coiled shards, the pores are aligned linearly (Figure 3a), in contrast to spiral shards (Figure 3b). However, the alignment is unlikely to be visible to the naked eye (Figure 3c,d), but can be identified with non-destructive testing [35]. Due to the size of the pores, alignments of the order of the millimeter, CT-scan [36], or synchrotron [37] were previously used but they are bulky and expansive. On the contrary, low-THz frequency radar-based techniques [38,39] provide compact systems while offering high lateral resolution within the diffraction limit of half a wavelength (typ. 1.5 mm at 100 GHz) [35,38,39].

However, when the issue to be solved is a binary classification, as in our case where we are trying to find out whether we have a spiral or coiled shard, this limit can be overcome by using AI-based classification and an appropriate measurement scheme. There are three well-known ways to increase measurement diversity, hence the resolution.

- The frequency diversity;
- The spatial diversity;
- The polarisation diversity.

As per spatial diversity, it depends on the transmitting and receiving angles with respect to the object. Multi-static measurements are commonly accepted as being more relevant for non-destructive evaluation because the wave goes through the object and, therefore, provides richer information. However, this significantly increases the system complexity. In addition, the shards typically range in depth from 8 mm to 1 cm. When imaging them with low-THz, the high frequency wave rapidly vanishes inside the clay medium from which the shards are made. This cancels out the advantage of multi-static measurements and we chose to work with mono-static measurements. There is a trade-off between spatial and frequency diversity that was clearly described in our previous paper [2]. The more frequencies, the fewer spatial measurement points for the classification accuracy close to 100% and vice-versa. Both scenarios have advantages and drawbacks:

- Working at a single frequency with a high number of antennas significantly reduces the complexity of the electronics at the component level since we do not need wide-band operation, but it is demanding in terms of the switching matrix.
- Working with a large bandwidth but a low number of antennas significantly reduces the complexity of the electronics at the system level because we get rid of the large switching matrix and the post-processing of the data coming from the various antennas, but requires wide-band components.

In this paper, we want to overcome this trade-off by pushing the co-optimization of the system and the AI algorithm to its ultimate limits. In other words, we aim at minimizing the number of antennas (measurement points) and frequencies far beyond what has been obtained before. In addition, we implement the possibility to choose between the vertical and horizontal polarizations for increasing flexibility. So, we start from the former obtained results [2] and consider two scenarios: in the first one, we use seven frequencies; in the second scenario, we use only one frequency. We use the spherical 3D scanner of our laboratory [40] for the measurements of the reflection coefficient of each shard over the entire D-band (i.e., 110–170 GHz) with a frequency step of 10 GHz over a scan area of θ × ϕ = ($20^{\circ }$× $20^{\circ }$), and with a scan step (denoted by $\Delta_{\theta ,\phi }$) of $0.2^{\circ }$. A total of 13 archaeological shards are used here. The total number of measurement points per shard and per frequency is 10201.

A database of classification samples is created by dividing the scan area into 51 patches, each consisting of 51 × 51 measurement points. The patches are created by shifting a window along the diagonal of the scan area by a scan step $\Delta_{\theta ,\phi }$ = $0.2^{\circ }$. Thus, each patch corresponds to the measurement of the shard at a particular incidence. From all these constraints on the measurement setup, the total number of measurement points is 5101, corresponding to 5101 sensors [2]. In the following, we will use the term ’image’ to refer to the matrix of measurement points obtained for each patch. The total number of data samples that we afford in the databases is derived as follows: for each shard, we afford 51 patches, multiplied by the number of frequencies, either 7 or 1. That is, for each shard, we afford

- A total of 357 classifier samples in the first scenario;
- A total of 51 classifier samples in the second scenario.

The validation database represents 10% of the training database. In Table 1 we summarize the characteristics of the classification database.

Throughout the paper, we use the False Recognition Rate (FRR) as a metric to judge the performance of the classifier. The FRR is extracted from the confusion matrix defined in Table 2. The FRR is defined in Equation (14).

(14) $$FRR=1-\frac{TN+TP}{TN+TP+FN+FP}$$

In Equation (14), the value of TN+TP+FN+FP is the number of data samples in the considered database. For the validation base this value is equal to 143 in Scenario $n^{\circ }$ 1, and to 21 in Scenario $n^{\circ }$ 2. For the test base, this value is equal to 3213 in Scenario $n^{\circ }$ 1, and to 459 in Scenario $n^{\circ }$ 2.

#### 2.2.3. Preliminary Study: Scan Step Maximization

Since our goal is to have a compact and mobile measurement system capable to detect the shape of the pores, we reduced the number of measurement points (sensors) by maximizing the scan step (denoted by $\Delta_{\theta ,\phi }$). This is carried out with the help of the continuous GWO algorithm, paying attention to the potential impact on the performance of the classifier. Reducing the number of sensors means that we are looking for the largest possible value ${\hat{\Delta }}_{\theta ,\phi }$ of the scan step $\Delta_{\theta ,\phi }$, which increases the ill-posedness of the inverse problem and thus decreases the ability of the classifier to distinguish between the shapes of the pores. We wish to retrieve the largest possible value of $\Delta_{\theta ,\phi }$ which yields a FRR value which is smaller than 0.2. This study has been presented in [2], though without any formal presentation. In Section 2.2.3, we set the problem as closed-form equations. The criterion, which is minimized by the GWO, is defined in Equation (15):

(15) C=FRR

with: 0 ≤C≤ 1. The criterion *C* is calculated with the validation database.

We aim at reaching a criterion value which is smaller than 0.2, as in [2]. So, in Section 2.2.3, two parameters are expected and estimated by the continuous GWO: the scan step $\Delta_{\theta ,\phi }$, and the number of neurons *L*.

The following notation holds: $x_{\alpha }\left(iter\right)={\left[x_{\alpha }^{1}\left(iter\right),x_{\alpha }^{2}\left(iter\right)\right]}^{T}$. The criterion *C* can also be denoted by $f({\left[x_{q}^{1}\left(iter\right),x_{q}^{2}\left(iter\right)\right]}^{T})$ when computed on wolf *q* at iteration iter. The convergence curve obtained by the GWO while minimizing the criterion in Equation (15) is defined by the following set of values:

(16) $$\left\{f\left({\left[x_{\alpha }^{1}\left(1\right),x_{\alpha }^{2}\left(1\right)\right]}^{T}\right),\ldots ,f\left({\left[x_{\alpha }^{1}\left(iter\right),x_{\alpha }^{2}\left(iter\right)\right]}^{T}\right),\ldots ,f\left({\left[x_{\alpha }^{1}\left(T_{max}\right),x_{\alpha }^{2}\left(T_{max}\right)\right]}^{T}\right)\right\}$$

Our purpose is to find, by a careful look at the values in Equation (16) and the values in $x_{\alpha }\left(iter\right)$, the largest instance $x_{\alpha }^{1}\left(\hat{iter}\right)$ of $x_{\alpha }^{1}\left(iter\right)$, which yields a criterion value *C* under 0.2. This step value, the largest possible, will be denoted by $\hat{\Delta_{\theta ,\phi }}$. We denote by $\hat{iter}$ the corresponding iteration index of the GWO. So, we define $\hat{iter}$ as

(17) $$\hat{iter}=max\left(\underset{iter}{argmax}\left\{x_{\alpha }^{1}\left(1\right),\ldots ,x_{\alpha }^{1}\left(iter\right),\ldots ,x_{\alpha }^{1}\left(T_{max}\right)\right\}\right)$$

submitted to

(18) $$f({\left[x_{\alpha }^{1}\left(iter\right),x_{\alpha }^{2}\left(iter\right)\right]}^{T})<0.2$$

In Equation (17), taking the max permits to remove any potential ambiguity, if more than one instance of iter yields the expected maximum step value, we choose the solution corresponding to the maximum value of iter. It corresponds, indeed, to the least possible value of false recognition rate as the values in the convergence curve decrease across the iterations. Solving Equation (17) yields $\hat{\Delta_{\theta ,\phi }}=3.2^{\circ }$ in Scenario $n^{\circ }$ 1, and $\hat{\Delta_{\theta ,\phi }}=1.2^{\circ }$ in Scenario $n^{\circ }$ 2. Therefore, in Scenario $n^{\circ }$ 1, the number of regularly spaced sensors is 37, the FRR value on the test database is 0.006224. In Scenario $n^{\circ }$ 2, the number of regularly spaced sensors is 217, and the FRR value on the test database is 0.037037. The GWO algorithm already allows us to reduce the number of sensor (from 5101 to either 37 or 217), with a minimum impact on the performance of the classifier (the mean FRR on the validation database is less than 0.2 in both scenarios). Despite these results, the step scan is still regular between two sensors, which is a drawback for our application and our goal. We will take these results into account in the criterion that is minimized by the CBTGWO, in Section 2.2.4.

#### 2.2.4. Sensor Selection and Neural Network Tuning: Definition of a Single Objective and Enumeration of the Parameters of Interest

Our aim is to switch the sensors on or off independently from each other, and to reduce the number of sensors which are switched on. Therefore, there is no longer any step value $\Delta_{\theta ,\phi }$ whose optimal value should be estimated. Instead, the state of each sensor should be estimated. In this subsection, the criterion that is minimized is as follows:

(19) $$C=\sqrt{\left(1+FRR\right)\left(1+\frac{S_{On}}{S}\right)}-1$$

where

- *S* is the number of sensors. We will set either S=37, when all frequencies are used or S=217, when one frequency is used;
- $S_{On}$ is the number of activated sensors, equivalently the number of sensors which are switched on;
- FRR is the false recognition rate obtained with the validation database.

The criterion *C* is a geometric mean, which permits to balance the influence of large values versus small values. We can notice that P=S+4: for i=1,…,S, $K^{i}$ is the state of sensor *i*, either 0 or 1, such that

(20) $$S_{On}=\sum_{i=1}^{i=S}K^{i}$$

For i=1,…,S, the values of $K^{i}$ are in a binary space: we switch each sensor either “ON” or “OFF” independently.

For i=S+1, $K^{i}=K^{S+1}$ is the polarization of the radar wave, either *H* (horizontal) or *V* (vertical).

For i=S+2, $K^{i}=K^{S+2}$ is the activation function, either relu (rectified linear unit), logistic, or tanh.

For i=S+3, $K^{i}=K^{S+3}$ is the solver, either adam (Adam adaptive moment estimation), sgd (stochastic gradient descent), or lbfgs (limited-memory Broyden–Fletcher–Goldfarb–Shanno).

For i=S+4, $K^{i}=K^{S+4}$ is the number of neurons *L* in the hidden layer. We have chosen three of the activation functions which are commonly used, and three well-performing solvers. As concerns the value of *L*, it cannot be smaller than 1, and we arbitrarily set its maximum possible value to 30.

The best possible case is when *C* is the closest possible to 1. We predict that *C* should probably be slightly higher than 1 because the value of $S_{On}$ which permits us to reach a zero-valued FRR, should be larger than 1 (or possibly equal to 1).

The interest of such a process is that once the optimal position and number of sensors being switched on is estimated, the other sensors are not needed. We also look for the best polarization of the scattering field. It is a parameter of our acquisition system which can be chosen as either horizontal or vertical. In parallel, we also look for the optimal MLP architecture by optimizing the number of neurons in the hidden layer, the solver, and the activation function. The expected parameters are either in binary, continuous, or ternary spaces. For the state of the sensors $\left\{OFF=0,ON=1\right\}$ and the polarization $\left\{H,V\right\}$ the search space is binary; the search space is ternary for the solver and for the activation function as well. A continuous search space is investigated with a continuous update rule for the number of neurons *L* in the hidden layer and so we used a floor to get an integer number. Table 3 summarizes the search space for all parameters.

In the next section, we propose a Continuous Binary Ternary GWO: it is a ‘mixed’ version of the GWO, which combines a continuous, a binary, and a ternary version of the GWO to tackle the problem of co-design presented here, and estimate the best values for all parameters presented in Table 3, either for the acquisition and for the processing of the radar data. This mixed method will be denoted by the CBTGWO (Continuous Binary Ternary GWO).

**Figure 4:**
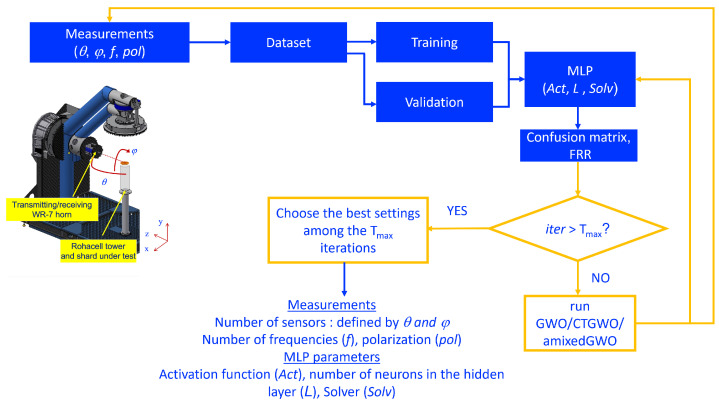
Workflow of the proposed method: training which consists in co-designing the acquisition and classification setup.

**Figure 5:**
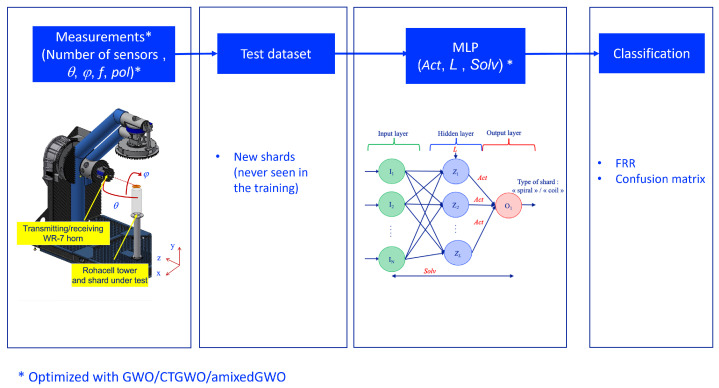
Workflow of the proposed method: test which consists in acquiring and classifying the data with the optimized parameters of the setup.

Considering all the materials described in this section, we end up with Figure 4 which describes the training process and with Figure 5 which describes the test process.

The update rule for the continuous and Ternary GWO are provided in Equations (1) and (13). The update rule that we propose for the Binary GWO is detailed in Section 3.

## 3. Methods: Proposed Continuous Binary Ternary GWO

We wish to preserve the original philosophy of the GWO: the number of leaders ruling the update of the agents is superior to 1, and the parameter *a* permits us to distinguish between an exploration phase at the beginning of the algorithm and an exploitation phase at the end. The continuous, original version of the GWO will be used to estimate the number of neurons in the hidden layer of the MLP because the search space is relatively wide (from 2 to 30); the ternary version proposed in [29] will be used to design the 2 other hyperparameters of the MLP. Indeed, data scientists usually compare the performances of three main solvers and three main activation functions to choose the best configuration for their application. Using an adaptive learning rate with adam can offer flexibility, but stochastic gradient descent performs well with relatively small datasets. As concerns lbfgs, it may outperform adam when the dataset is relatively small. See [41,42,43] and references inside. As the dataset considered in this paper is relatively small, we decide to take these three solvers as candidates.

We propose for the first time in this paper a binary version to design the sensor system, which favors the 0 value.

### 3.1. Novel Adaptive and Chaotic Expression of *a*

In this work, we combine the expression proposed in [13] and the expression proposed in [29], which yield an adaptive and chaotic version of *a*:

(21) a=2(1−a2(0.5+Γ(q,iter)))

where Γ(q,iter) is a value taken from a chaotic sequence as in [29], and a2 is defined as follows:

(22) $$a2=\left\{\begin{array}{ccc} {\left(iter/\left(T_{max}/2\right)\right)}^{\eta } & if & iter\leq T_{max}/2\\ {\left(\left(iter-T_{max}/2\right)/\left(T_{max}/2\right)\right)}^{1/\eta } & otherwise \end{array}\right.$$

with η=2, and assuming $T_{max}$ is even.

### 3.2. Novel Binary GWO Favoring 0 Values

In the considered co-design problem, we face a constraint: the method that is meant to select the sensors which are switched on or off should favor the sensor switch off. That is why the proposed novel Binary GWO still explores a binary search space but favors 0 values.

#### 3.2.1. Contribution of a Leader

As explained previously in the paper, in the case where the search space is continuous, the contribution $y_{l}^{i}\left(iter\right)$ corresponding to any leader *l* is calculated through Equation (5). In Equation (5), the contribution $y_{l}^{i}\left(iter\right)$ may be smaller than 0 and larger than 1. So, we enforce clip this value in this interval:

(23) $$y_{l}^{i}(iter)=clip(x_{l}^{i}-b^{i}d_{l}^{i}(iter))$$

where $b^{i}$ is defined as in Equation (3) and $d_{l}^{i}(iter)$ is defined as in Equation (4); the clip consists in setting a value which is lower than 0 to 0 and a value which is larger than 1 to 1.

#### 3.2.2. Binary Update Rule

In the considered issue of sensor selection, we wish to favor the selection of the ‘off’ state for the largest possible number of sensors. That is, we wish to favor the value 0 while updating any wolf position. In the proposed novel version of the Binary GWO, wolf *q* is updated from iteration iter to iteration iter+1 as follows:

$x_{q}^{i}\left(iter+1\right)=$

(24) $$\;\left\{\begin{array}{ccc} 1 & if & r\leq \varphi \frac{y_{\alpha }^{i}(iter)+y_{\beta }^{i}(iter)}{2}c1(a)c2(a)\\ 0 & otherwise \end{array}\right.$$

Equation (24) is a modified version of Equation (22), where the slope of the Sigmoid is set to a real-valued scalar c1(a) and the inflexion point of the Sigmoid is set to a real-valued scalar c2(a).

We propose the following expressions for c1(a) and c2(a):

(25) c1(a)=30(1−a/3)

and

(26) c2(a)=0.75+0.1a

The interest of these rules, compared to the update rules in Equation (22), is two-fold:

- This emphasizes the exploration capacities of the method at the beginning of the algorithm, because c1(a) is small at the beginning and value 0 may be chosen even for large values of *y*;
- This permits us to favor the choice of the value 0, because c2(a)>0.5.

**Figure 6:**
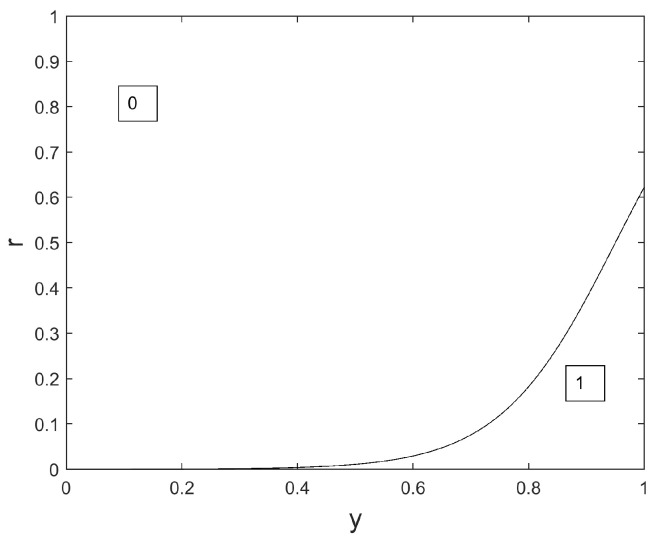
Binary map privileging 0 values, iter=0, a=2.

**Figure 7:**
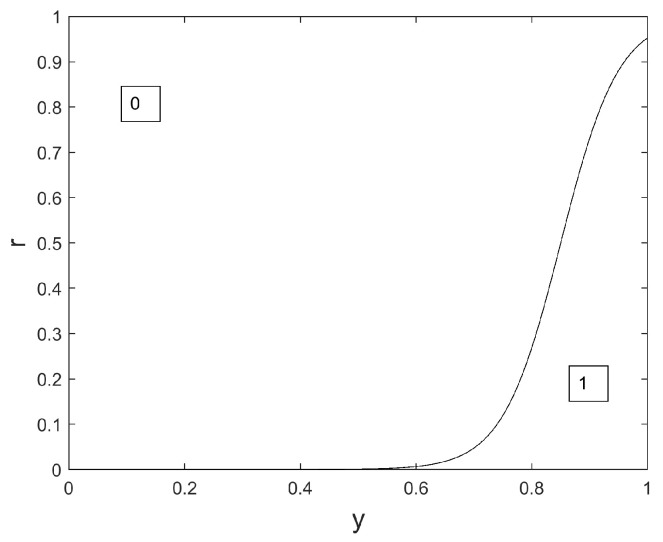
Binary map privileging 0 values, $iter=\frac{T_{max}}{2}$, a=1.

**Figure 8:**
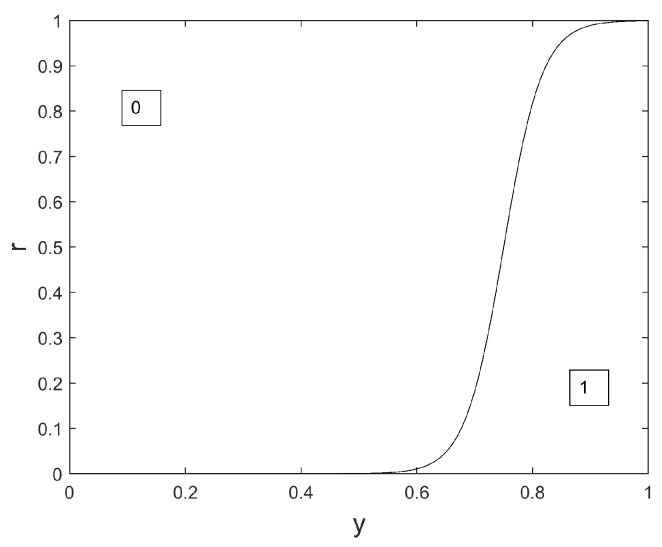
Binary map privileging 0 values, $iter=T_{max}$, a=0.

This novel version is illustrated in Figure 6, Figure 7 and Figure 8.

We notice in Figure 6, Figure 7 and Figure 8 that the surface dedicated to 0 is increased, compared to the original Binary GWO (see Figure 1), whatever the iteration index. Consequently, the probability to choose 0 for a given value of $\frac{y_{\alpha }^{i}(iter)+y_{\beta }^{i}(iter)}{2}$ is also increased whatever the iteration index. This is of great interest for our application where a large number of sensors should be switched off, and an important novelty compared to previous works such as the original Binary GWO proposed in [31], and the Ternary GWO proposed in [29]. Another novelty, compared to the binary versions presented in [31,44], is the ‘evolutive’ nature of our version of the Binary GWO: at the beginning of the optimization process, for small values of iter, it is possible for the value 0 to be selected, even if the two leaders, for parameter *i*, bear the value 1. See the illustration in Figure 6. If $\frac{y_{\alpha }^{i}(iter)+y_{\beta }^{i}(iter)}{2}$, the value 0 is selected with a probability of 0.38 at iteration iter=0, with a probability of 0.05 at iteration $iter=T_{max}/2$, with a probability almost equal to 0 at iteration $iter=T_{max}$.

This evolutive nature enhances the exploration abilities of the proposed method at the beginning of the process.

### 3.3. Pseudo-Code CBTGWO (Algorithm 1)

We propose combining the continuous and ternary versions mentioned in Section 2 with the proposed binary version of the GWO, which privileges the choice of value 0. A novelty with respect to the adaptive mixed GWO proposed in [13] is the combination of three update rules instead of two, repositioning of the three worst agents at Step 4, and inserting a memory effect at Step 6: an agent is updated if the new score is better than the previous one.
**Algorithm 1:** Pseudo-code: Continuous Binary Ternary Grey Wolf Optimization**Inputs**: fitness function, number of *Q* search agent, search space of *P* parameters, maximum of iteration $T_{max}$, small factor ϵ set by the user, to stop the algorithm.Set iteration number iter=1, create an initial herd composed of *Q* wolves with all required parameter values $x_{q}iter$, q=1,…,Q. This initial population forms of a matrix with *Q* rows and *P* columns.Evaluate fitness function value $C(x_{q}iter)$ of each wolf $x_{q}iter$, q=1,…,Q.Sort the wolves through their fitness value and update the α, β, and δ wolves which hold, respectively, the first, second, and third best fitness value. Store their position in vectors $x_{\alpha }iter$, $x_{\beta }iter$, and $x_{\delta }iter$, respectively.Reposition the three worst agents which become$\frac{x_{\alpha }\left(iter\right)+x_{\beta }\left(iter\right)}{2}$, $\frac{x_{\alpha }\left(iter\right)+x_{\delta }\left(iter\right)}{2}$, and $\frac{x_{\beta }\left(iter\right)+x_{\delta }\left(iter\right)}{2}$, respectively.Repeat steps for each wolf $x_{q}\left(iter\right)$, q=1,…,Q:For each component $x_{q}(iter)$ with i=1,…,P:compute $x_{q}^{i}\left(iter+1\right)$ using:(a)Equation (24) if $K_{i}$ takes its values in a binary search space;(b)Equation (13) if $K_{i}$ takes its values in a ternary search space;(c)Equation (6) if $K_{i}$ takes its values in a continuous search space.if $C(x_{q}(iter+1))$<$C(x_{q}\left(iter\right))$ update $x_{q}\left(iter\right)$ as $x_{q}(iter+1)$.Exchange the current population with the new one, obtained at step 5.If $iter<T_{max}$ or $C(x_{q}(iter))>\epsilon$, increase iter, and go to step 2.**Output**: estimated parameter values $\hat{K_{1}},\hat{K_{2}},\ldots ,\hat{K_{P}}$

In Section 4, we aim, firstly, to illustrate the performances of the CBTGWO on a synthetic test function with the same number of parameters as in the considered co-design application. Secondly, we evaluate our method and comparative optimization algorithms on experimental radar data acquired from shards.

## 4. Results

In Section 4.1, we describe the experimental conditions in terms of software, metrics, and comparative methods. The expressions and optimal solutions of the surrogate function, which are based on a paired of widely used synthetic functions, are also detailed in this subsection. In Section 4.2 we present a case where we afford 37 sensors and several frequencies of the D-band and a case where we afford 217 sensors and only one frequency.

### 4.1. Performance Evaluation on a Surrogate Function

In order to evaluate the performances of the proposed CBTGWO, a synthetic function which is supposed to adequately represent the proposed problem is considered. The continuous version is used for one parameter, the ternary version for two parameters, and the binary version for all other parameters.

#### 4.1.1. Experimental Conditions and Metrics

In this section, the test environment is a Win10 flagship 64-bit operating system with a dual-core Intel Core i5-4210 M @2.60 GHz and 16 GB internal storage.

The software is Python 3.7.0. As comparative methods, we use the adaptive mixed GWO in discrete mode [13] (denoted by amixedGWO), and the vanilla continuous GWO [12]. We run all methods with Q=50 agents and $T_{max}=300$ iterations. The agents are initialized at random, with values between the lower and upper bounds of the search spaces. Refer to Table 3: the upper bound is 1 for the binary search space, 2 for the ternary search spaces, and 30 for the continuous search space; the lower bound is 0 for all search spaces. The algorithms are run R=30 times for each test function to get statistical results.

The statistical performances of the algorithms are computed and displayed in terms of geometric average (GAvg) of the final score and of the convergence curves:

(27) $$GAvg={\left(\prod_{\rho =1}^{R}C{\left(x_{\alpha }(T_{max})\right)}_{\rho }\right)}^{\frac{1}{R}}$$

where ρ denotes the index of the run, *R* the number of runs, and $C{\left(x_{\alpha }(T_{max})\right)}_{\rho }$ the score obtained at run ρ.

#### 4.1.2. Description of the Benchmark Functions

Functions $F_{1}$ and $F_{2}$ are defined in Table 4. $F_{1}$ is unimodal and $F_{2}$ is multimodal. In Table 4, *P* denotes the dimension. Vector $x={\left[K^{1},K^{2},\ldots ,K^{P}\right]}^{T}$ is a set of input parameters to any test function.

We define the criterion C(x) depending on *P* values in vector x, as

(28) $$C\left(x\right)=\sqrt{\left(1+F_{1}\left(x-x_{min}\right)/P\right)\left(1+\sqrt{F_{2}\left(x-x_{min}\right)}/P\right)}-1+o$$

We reiterate that *P* in Equation (28) is the number of parameters to be estimated. $F_{1}/P$ is a surrogate for the false recognition rate, and $\sqrt{F_{2}}/P$ is a surrogate for the proportion of sensors which are turned on. We set $o=10^{-8}$, which is the minimum value of criterion *C*, reached at location $x_{min}$.

#### 4.1.3. Experiments

We set the position of the expected global minimum as $x_{min}=[0\;,\ldots ,1\;,\ldots ,0\;,H,relu$, ${lbfgs,2]}^{T}$. That is, we expect a single sensor switched ON, polarization *H*, the first activation function, the third solver function, and two neurons.

**Figure 9:**
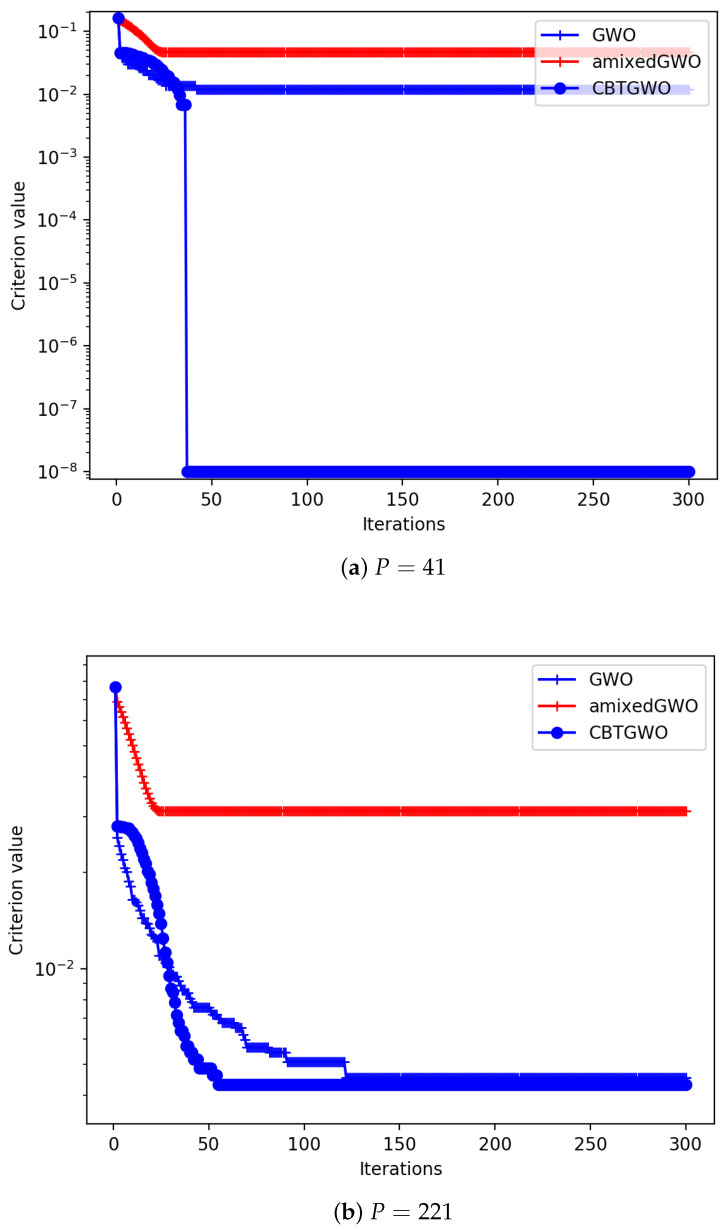
Minimization of criterion *C* defined in Equation (28) with CBTGWO, amixedGWO, and GWO: experiment with 50 agents, 300 iterations, P=41 and 221.

Experiments are performed for which we display the geometric average of the convergence curve, that is, the score $C(x_{\alpha }iter)$ across iterations. We have performed some experiments with small values of *P* such as P=10 and P=20. We could notice that the CBTGWO and amixedGWO behave well in the case where P=10. With this relatively small number of parameters, the amixedGWO even outperforms slightly the CBTGWO and GWO. When P=20, the amixedGWO no longer outperforms the CBTGWO and GWO. The most interesting results concern the values of *P* which simulate the presence of 37 and 217 sensors, that is, P=41 and P=221. These results are displayed in Figure 9. In the case where P=41, the proposed method reaches the expected value $10^{-8}$; the GWO yields $2.5\times 10^{-2}$ and the amixedGWO $6\times 10^{-2}$.

In the case where P=221, the proposed method yields $GAvg=4.3\times 10^{-3}$; the GWO yields $GAvg=4.5\times 10^{-3}$ and amixedGWO $GAvg=3.1\times 10^{-2}$. Due to the high dimensionality of the problem, the proposed method does not reach the expected minimum, but still outperforms the comparative methods. Whatever the value of *P*, these results illustrate the exploratory abilities of the CBTGWO: the convergence curve values slowly decrease across the first iterations, and the CBTGWO eventually reaches an average score value GAvg which is smaller than the score obtained by the GWO or amixedGWO.

Although function *F* in Equation (28) is only a ’surrogate’ for the considered co-design problem, the simulation results show that the proposed CBTGWO is trustworthy. We investigate its performances on real-world data in Section 4.2.

### 4.2. Results Obtained on Real-World Radar Data

#### 4.2.1. Minimization of the Number of Sensors Operating at All D-Band Frequencies

For the first experiment, we aimed to reduce the number of sensors which are switched on among the S=37 sensors operating in the D-band and to keep only the most relevant sensors for the classification. We set the number of search agents as Q=10 and the maximum number of iterations as $T_{max}=100$.

Theoretically, the best solution is reached with $S_{On}$ being equal to 1. From physical considerations, we predict that this single sensor should be placed vertically or close to the vertical. The theoretical least possible value $C_{min}$ of the criterion is:

(29) $$C_{min}=\sqrt{\left(1+0\right)\left(1+\frac{1}{37}\right)}-1=0.013423419$$

**Figure 10:**
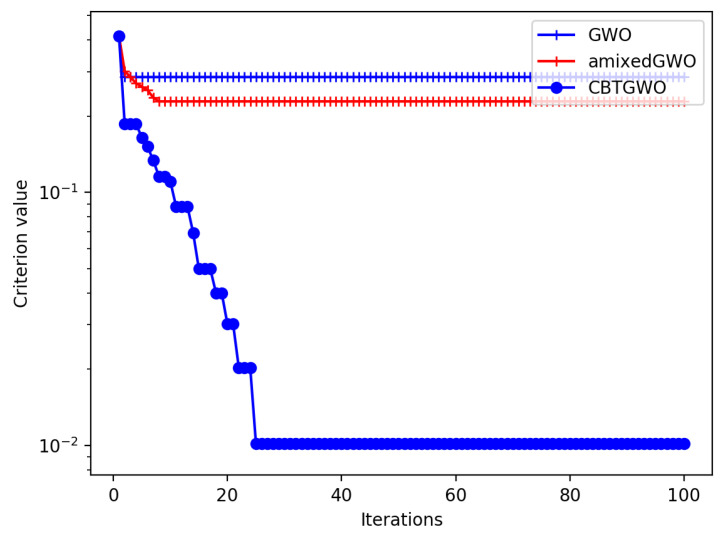
Minimization of criterion *C* defined in Equation (19): convergence curve of CBTGWO, GWO, and amixedGWO algorithms for Scenario $n^{\circ }$1.

In Figure 10, we present the convergence curve of the three optimization methods. It is worth noticing that, contrary to the convergence curves in Figure 9, the curves should not reach $10^{-8}$ but $C_{min}=0.013423419$.

It can be observed that the convergence curve of the CBTGWO decreases faster than the curves of the amixedGWO and the GWO. The results of the optimization are as follows.

As concerns the CBTGWO, the final experimental value $C(x_{\alpha }(T_{max}))$ of the criterion is equal to the best possible value 0.013423419 defined in Equation (29) for the CBTGWO, which yields $S_{On}=1$ sensor and FRR = 0.

As concerns the GWO, it has reached a final value $C(x_{\alpha }(T_{max}))$ = 0.285714285, with $S_{On}=8$ sensor and FRR = 0.421052631.

As concerns the amixedGWO, it has reached a final value $C(x_{\alpha }(T_{max}))$ = 0.228903609, with $S_{On}=25$ sensor and FRR = 0.0. We assume that the GWO and the amixedGWO algorithms have reached a local minimum, and not the global one.

In some previous works using the amixedGWO [13] the number of estimated parameters is significantly smaller than in the study of this paper, which may have been an advantage for the amixedGWO.

The amixedGWO yields a 0-valued FRR but the CBTGWO algorithm provides a number of neurons which yields a 0-valued FRR with only 1 sensor instead of 25.

**Figure 11:**
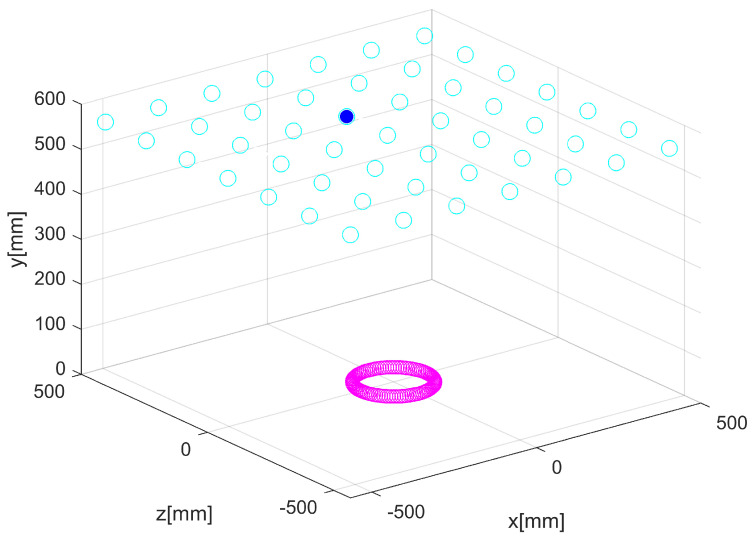
Optimization of the number of sensors for Scenario $n^{\circ }$1: CBTGWO. Empty cyan circles denote the potential positions of the sensors. The full blue circle denotes the position of the selected switched on sensor.

**Figure 12:**
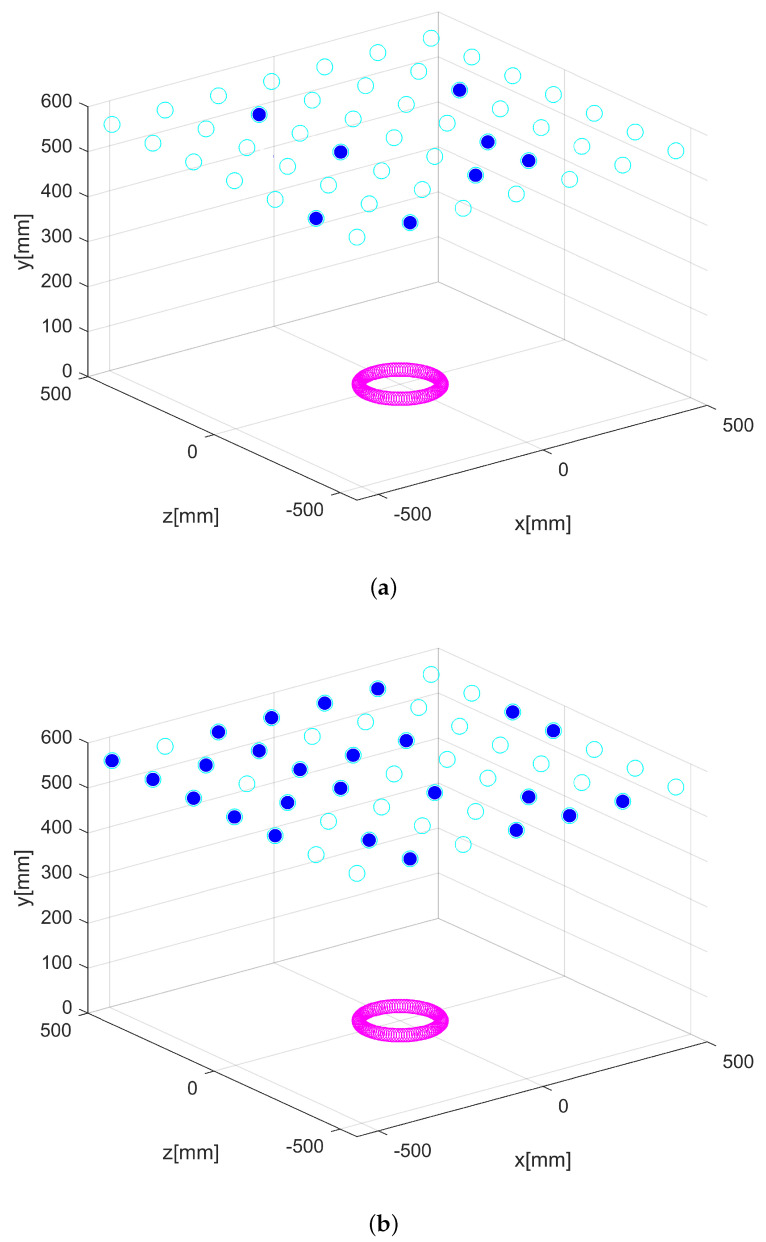
Optimization of the number of sensors for Scenario $n^{\circ }$1: GWO (**a**) and amixedGWO (**b**). Full blue circles denote the position of the selected switched on sensors.

At the last iteration of the CBTGWO algorithm, we obtained:

$$\begin{array}{ccc} C(x_{\alpha }(T_{max}))=0.013423419 & \; & x_{\alpha }(T_{max})={\left[K^{1},\ldots ,K^{19},\ldots ,K^{37},H,relu,adam,15\right]}^{T} \end{array}$$

where $K^{19}=1$ and $K^{i}=0$∀i≠19. The best-measured field is with horizontal polarization, the number of neurons in the hidden layer is $\hat{L}$ = 15, the activation function is relu, and the solver function is adam. The computational time is 4628 s. For the three optimization methods, the optimal location of the sensor is shown in Figure 11 and Figure 12.

Table 5 and Table 6 summarize the results obtained by the three optimization methods. The acquisition time is provided for IF = 100 Hz. We notice that the optimization time is about 2 times higher for the CBTGWO (essentially due to the memory effect) but the acquisition time, once the setup is designed, is 8 and 25 times smaller compared to the GWO and amixedGWO, respectively.

After the optimization and training phases, we classified the 9 shards of the test database and got an FRR value equal to 0.003112356. That is, 10 data samples out of 3213 are not correctly classified.

The design of this system operating at the seven frequencies of the D-Band is feasible but still complex. We propose another alternative in Section 4.2.2.

#### 4.2.2. Minimization of the Number of Sensors Operating at the Central D-Band Frequency

**Figure 13:**
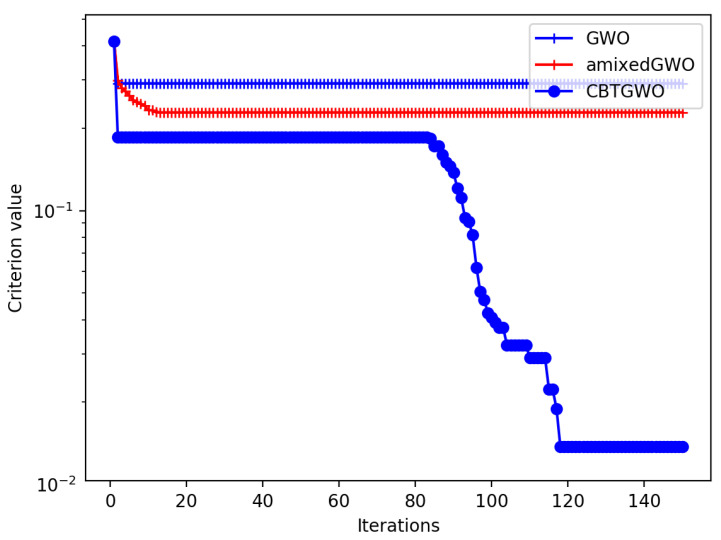
Minimization of criterion *C* defined in Equation (19): convergence curve of the CBTGWO, GWO, and amixedGWO algorithms for Scenario $n^{\circ }$ 2.

Frequency diversity is very important for our application, but the design and development of a compact measurement system operating over the entire D-band is complex. Therefore, we need to drastically reduce the number of sensors but also the frequency bandwidth. The spatial diversity is more important and crucial when the frequency diversity is reduced. Therefore, we propose to reduce the number of “ON” sensors among the S=217 sensors operating at the central frequency of the D-band (140 GHz). We use the criterion defined in Equation (29). We do not expect $S_{On}$ to be equal to 1. Indeed, the characterization of the pores with one frequency needs some spatial diversity. As in the first experiment, we are also estimating the best values for the polarization, the number of neurons in the hidden layer, the activation function, and the solver. The number of search agents is Q=50 and the maximum number of iterations is $T_{max}=150$. The convergence curves of the CBTGWO, GWO and amixedGWO algorithms are shown in Figure 13. The convergence curve of the CBTGWO decreases faster than the convergence curve of the comparative methods, and the best score, $C(x_{\alpha }(T_{max}))=0.014897307$, is provided by the CBTGWO. The score achieved by the CTBGWO can be expressed as follows:

(30) $$\begin{array}{c} C=\sqrt{\left(1+0.02061\right)\left(1+\frac{2}{217}\right)}-1=0.014897307 \end{array}$$

That is, we reach an FRR value on the validation base which is equal to 0.02061 with $S_{On}=2$ sensors which are switched on. With the GWO and amixedGWO, the final value $C(x_{\alpha }(T_{max}))$ is equal to 0.291441082 and 0.228271353, respectively. Even though the amixedGWO provides a lower FRR value, the value of $S_{ON}$ is much higher than with the CBTGWO. Again, the CBTGWO provides a good compromise between the number of sensors and FRR (which is only 2%). As for the Scenario $n^{\circ }$ 1, the GWO and amixedGWO algorithm have found a solution corresponding to a local minimum.

At the last iteration of the CBTGWO algorithm,

$$\begin{array}{c} C(x_{\alpha }(T_{max}))=0.014897307 \end{array}$$

and

$$\begin{array}{c} \alpha T_{max}={\left[K^{1},\ldots ,K^{47},\ldots ,K^{109},\ldots ,K^{217},V,relu,adam,28\right]}^{T} \end{array}$$

where $K^{47}=K^{109}=1$ and $K^{i}=0$∀i≠47,109. The components of the wolf α do not change after iteration 119.

Table 7 and Table 8 summarize the results obtained by the three optimization methods. The acquisition time is provided for IF = 100 Hz.

**Figure 14:**
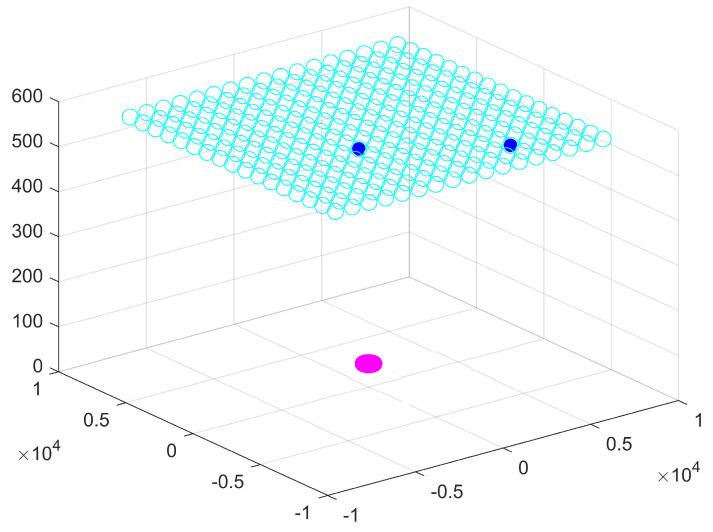
Optimization of the number of sensors for Scenario $n^{\circ }$2: CBTGWO. Empty cyan circles denote the potential positions of the sensors. Full blue circles denote the position of the selected switched on sensors.

**Figure 15:**
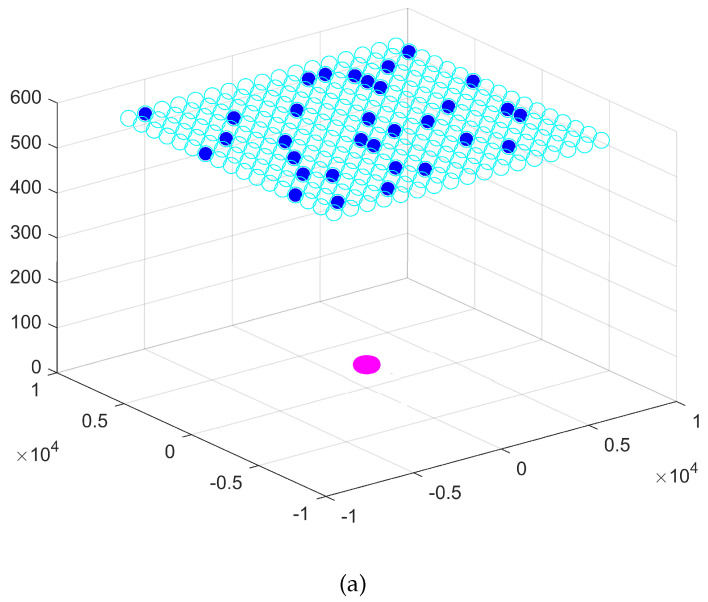

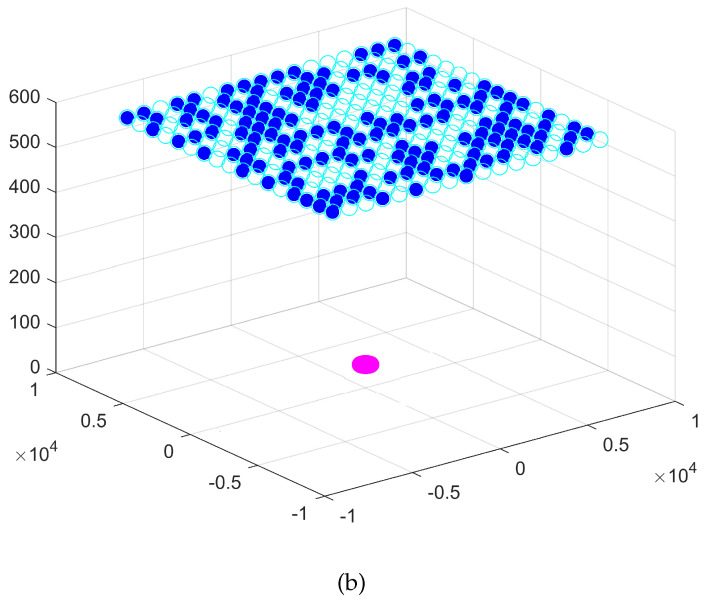
Optimization of the number of sensors for Scenario $n^{\circ }$2: GWO (**a**) and amixedGWO (**b**). Empty cyan circles denote the potential positions of the sensors. Full blue circles denote the position of the selected switched on sensors.

For the CBTGWO, the best-measured field is with vertical polarization. The number of neurons in the hidden layer is $\hat{L}=28$, the activation function is relu, and the solver function is adam (see Table 7). The computational time is 6559 s for the CBTGWO, 3055 s for the GWO, and 3426 s for the amixedGWO. So, we notice that, as in the case with 37 sensors, the optimization time is about 2 times higher for the CBTGWO. However, the CBTGWO yields a measurement time which is, by far, the smallest. The obtained optimal sensor location is shown in Figure 14 and Figure 15.

This configuration yields a measurement time of 5.4 s for the configuration provided by the CBTGWO, 86.4 s for the configuration provided by the GWO, and 396.9 s for the configuration provided by the amixedGWO (see Table 8). The convergence curves in Figure 13 illustrate the exploration abilities of the proposed CBTGWO: the convergence curve of the reaches a minimum which is 20 to 30 times smaller than with the two comparative methods, which have missed the global minimum. This illustrates the exploration abilities of the proposed algorithm, possibly due to the adaptive and chaotic nature of the expression of *a* that we propose in this paper (see Equation (21)). Moreover, the solution which is proposed by the CBTGWO is consistent with the physical considerations: the locations of the two selected sensors in Figure 14 offer two points of view which are complementary. Hence, the small value of the FRR. The CBTGWO offers an MLP architecture which handles the data provided by only two sensors: it yields an FRR value on the validation base which is smaller than with the GWO, with a smaller number of sensors. The amixedGWO yields a smaller FRR value, but with a number of sensors and a computational time for the acquisition which is 73 times higher (see Table 8).

We classify that the 9 shards included in the test database and the FRR obtained on this test database are equal to 0.023965141. That is, 11 images out of 459 are not correctly classified.

We also performed another experiment which consists of keeping the same sensors and the same MLP architecture with its optimal parameters found previously and changing only the working frequency. We obtained that, whatever the value of the frequency, the FRR is less than 3%. These interesting results mean that we do not need to optimize the location and the number of sensors and the MLP architecture if the working frequency is changed. In this case, the spatial diversity provides more information than the frequency diversity.

## 5. Discussion

The mixed optimizer composed of the continuous GWO, the Ternary GWO, and the Binary GWO solve the considered problem of co-design of a radar system and a neural network for experimental radar data processing. Numerical simulations performed on a surrogate benchmark function show the good behavior of the proposed CBTGWO with respect to the vanilla GWO proposed in [12] and the discrete version proposed in [13]. We show that when 37 sensors are simulated, the exact location of the global minimum of the surrogate function is found by the proposed CBTGWO.

This justifies its use in the considered co-design problem: we optimize the number and the location of a set of sensors, and jointly tune the parameters of a multilayer perceptron to perform shard classification from millimeter-wave measurements.

In a wide-band scenario, using the vanilla GWO and a fixed step between the sensors, we reach a 8.3% FRR on the validation base with 37 sensors; using the proposed CBTGWO, we reach a 0% FRR on the validation base with only 1 sensor. On the test database, 10 images out of 3213 are not correctly classified.

In a scenario with only one frequency, using the vanilla GWO and a fixed step between the sensors, we reach a 0% FRR on the validation base with 217 sensors; using the proposed CBTGWO we reach a 1.3% FRR on the validation base with only 2 sensors. On the test database, 11 images out of the 459 images are not correctly classified. The results obtained in the wide-band scenario point out a limitation which is inherent to the classification process: whatever the performance of the optimization method, a test database is always different from a validation database. We infer from these results that the appropriate strategy consists in finding a trade-off between the FRR value obtained on the validation database and the number of sensors, instead of aiming at the least possible FRR value on the validation database.

## 6. Conclusions

The contributions of this paper are three-fold. Firstly, we set a problem of radar system design, in which the number of sensors which are turned on should be minimized, and a false recognition rate should also be as small as possible. Secondly, we propose a novel binary version of the Grey Wolf Optimizer, which favors the choice of the value 0 and sensor switch off; this Binary GWO, combined with the Ternary Grey Wolf Optimizer and the continuous GWO, yield the CBTGWO. Thirdly, we apply our CBTGWO to perform the co-design of a radar acquisition system, jointly with the classification neural network. We thereby solve a major issue in the radar community, which is the selection of the least and most relevant sensors for a given application. In a wide-band scenario, the proposed method manages to select only one sensor while yielding a zero-valued FRR. In a scenario with only one frequency, three sensors are selected and placed in a manner that preserves the spatial diversity. Future research could consider other classification issues, on data acquired in a different manner. The proposed methodology could be used to design other sensor architectures (in different wavelength domains, for instance), and other neural networks including, for instance, convolutional layers.

## Figures and Tables

**Table 1:** Classifier database: number of classifier samples for each scenario.

Scenario	Total per Shard	Training	Validation	Test	Total
$n^{\circ }$ 1: 7 frequencies	357	1285	143	3213	4641
$n^{\circ }$ 2: 1 frequency	51	183	21	459	663

**Table 2:** Definition of the confusion matrix.

Classes	Negative (Actual)	Positive (Actual)
**Negative (Predicted)**	TN: True Negative	FN: False Negative
**Positive (Predicted)**	FP: False Positive	TP: True Positive

**Table 3:** Radar system and MLP configuration: jointly optimized parameters and search spaces.

Optimized Component	Parameter	Search Space Category	Range
Radar system	Sensor state	Binary	$\left\{OFF,ON\right\}$
	Polarization		$\left\{H,V\right\}$
MLP configuration	Activation function	Ternary	$\left\{relu,logistic,tanh\right\}$
	Solver function		$\left\{adam,sgd,lbfgs\right\}$
	Number of neurons	Continuous	[1:30]

**Table 4:** Synthetic benchmark functions.

Function
$F_{1}\left(x\right)=-20\exp\left(-0.2\sqrt{\frac{1}{P}\sum_{i=1}^{P}{\left(10K^{i}\right)}^{P}}\right)-\exp\left(\frac{1}{P}\sum_{i=1}^{P}\cos\left(20\pi K^{i}\right)\right)+20+e$
$F_{2}\left(x\right)=\sum_{i=1}^{P}{K^{i}}^{2}$

**Table 5:** Scenario $n^{\circ }$1. Summary of the results for the tree optimization methods: CBTGWO, GWO, and amixedGWO.

Methods	$C(x_{\alpha }(T_{max}))$	FRR	$S_{\mathrm{ON}}$	Act.	Solv.	Pol.	*L*
CBTGWO	0.013423419	0	1	Relu	adam	H	15
GWO	0.285714285	0.4210	8	Relu	adam	H	15
amixedGWO	0.228903609	0.0249	25	Relu	lbfgs	H	9

**Table 6:** Scenario $n^{\circ }$1. Computational time for the optimization, and for the optimized acquisition: CBTGWO, GWO, and amixedGWO.

Methods	Optimization Time	Acquisition Time
CBTGWO	4628 s	3.2 s
GWO	2184 s	25.6 s
amixedGWO	2063 s	80 s

**Table 7:** Scenario $n^{\circ }$2. Summary of the results for the tree optimization methods: CBTGWO, GWO, and amixedGWO.

Methods	$C(x_{\alpha }(T_{max}))$	FRR	$S_{\mathrm{ON}}$	Act.	Solv.	Pol.	*L*
CBTGWO	0.014897307	0.0206	2	Relu	adam	V	28
GWO	0.291441082	0.5015	32	Logistic	lbfgs	H	16
amixedGWO	0.228271353	0.0	147	tanh	lbfgs	V	27

**Table 8:** Scenario $n^{\circ }$2. Computational time for the optimization, and for the optimized acquisition: CBTGWO, GWO, and amixedGWO.

Methods	Optimization Time	Acquisition Time
CBTGWO	6559 s	5.4 s
GWO	3055 s	86.4 s
amixedGWO	3426 s	396.9 s

## Data Availability

Data are contained within the article.

## Abbreviations

The following abbreviations are used in this manuscript:

GWO	Grey Wolf Optimizer
CBTGWO	Continuous Binary Ternary Grey Wolf Optimizer
MLP	Multilayer Perceptron
*lbfgs*	Limited-Memory Broyden–Fletcher–Goldfarb–Shanno
*sgd*	Stochastic Gradient Descent
*adam*	Adaptive Moment Estimation
